# Establishment of human trophoblast stem cells from human induced pluripotent stem cell-derived cystic cells under micromesh culture

**DOI:** 10.1186/s13287-019-1339-1

**Published:** 2019-08-07

**Authors:** Zhuosi Li, Osamu Kurosawa, Hiroo Iwata

**Affiliations:** 1 The “Compass to Healthy Life” Research Complex Program, RIKEN Institute, Kobe, 650-0047 Japan; 2 Research Promotion Institution for COI Site, Kyoto University, Kyoto, Japan

**Keywords:** Micromesh, Trophoblast, Human pluripotent stem cell, Syncytiotrophoblast, Extravillous trophoblast

## Abstract

**Background:**

Trophoblasts as a specific cell lineage are crucial for the correct function of the placenta. Human trophoblast stem cells (hTSCs) are a proliferative population that can differentiate into syncytiotrophoblasts and extravillous cytotrophoblasts. Many studies have reported that chemical supplements induce the differentiation of trophoblasts from human induced pluripotent stem cells (hiPSCs). However, there have been no reports of the establishment of proliferative hTSCs from hiPSCs. Our previous report showed that culturing hiPSCs on micromesh as a bioscaffold induced cystic cells with trophoblast properties. Here, we aimed to establish hTSCs from hiPSCs.

**Methods:**

We used the micromesh culture technique to induce hiPSC differentiation into trophoblast cysts. We then reseeded and purified cystic cells.

**Results:**

The cells derived from the reseeded cysts were highly proliferative. Low expression levels of pluripotency genes and high expression levels of TSC-specific genes were detected in proliferative cells. The cells could be passaged, and further directional differentiation into syncytiotrophoblast- and extravillous cytotrophoblast-like cells was confirmed by marker expression and hormone secretion.

**Conclusions:**

We established hiPSC-derived hTSCs, which may be applicable for studying the functions of trophoblasts and the placenta. Our experimental system may provide useful tools for understanding the pathogenesis of infertility owing to trophoblast defects in the future.

**Electronic supplementary material:**

The online version of this article (10.1186/s13287-019-1339-1) contains supplementary material, which is available to authorized users.

## Background

Trophoblasts play key roles in the placenta. Placentation starts with the formation of the trophectoderm in pre-implantation blastocysts. After implantation, trophectoderm cells differentiate into diverse trophoblast cell types. Mononuclear cytotrophoblasts (CTBs), derived from the trophectodermal layer, are considered to be trophoblast stem cells (TSCs) and differentiate into either syncytiotrophoblasts (STBs) or extravillous trophoblasts (EVTs) [1]. STBs comprise the cell layer that covers the outer surface of the villi and are responsible for nutrient and gas exchange in the human placenta [2]. Villous STBs are also the major site for the production of numerous hormones, such as human chorionic gonadotropin (hCG). For the maintenance of pregnancy and immunomodulation, hCG needs to be delivered to the maternal side and circulated in the body [3, 4]. TSCs differentiate into EVTs at villus/uterus anchoring sites. EVTs begin to migrate into the decidual tissue or toward maternal blood vessels. This invasion causes maternal arteria to lose contractility and maintain sufficient maternal blood flow in intervillous spaces to support placental function [5]. Several reports have demonstrated that inadequate formation of the STB structure or failure of EVT invasion leads to severe pregnancy disorders, such as preeclampsia [6].

Various cell types have been used in placental research. Human choriocarcinoma cell lines, such as BeWo and JEG-3, have been widely used as models for mimicking in vitro trophoblast cell fusion in the presence of cyclic adenosine monophosphate (cAMP)-elevating agents, such as forskolin or 8-bromo-cAMP [7, 8]. Primary cells isolated and purified from the human term placenta have been used to obtain functional STBs [9]. Okae et al. [10] have successfully isolated and maintained human TSC (hTSC) lines from first-trimester placentas and have shown that these hTSCs have the capacity to differentiate into STBs and EVTs. However, because of ethical problems, it is difficult to acquire human placental tissues and blastocysts in order to isolate cells.

According to recent reports, human induced pluripotent stem cells (hiPSCs) and human embryonic stem cells (hESCs) can be induced into trophoblast lines using culture medium with added bone morphogenetic protein 4 (BMP4) and without fibroblast growth factor-2 (FGF2) [11–14]. BMP4 has been reported to induce CTBs that cannot proliferate but can nondirectionally differentiate into STBs and EVTs in the same culture system [15].

Bioscaffolds, such as collagen, silk, and nanofibers, are increasingly used to promote the differentiation of hiPSCs and the maintenance of cellular physiological functions [16, 17]. We originally developed a cell culture technique with a microstructured mesh sheet as a bioscaffold. The apertures of micromeshes commonly used for culture are much larger in size than single cells, allowing cells present at the openings to become to be bound only by cell-cell adhesion [18]. Minimization of cell-basal substrate adhesion can promote cellular functions and is beneficial for cells to obtain oxygen and nutrients. Notably, micromesh-cultured hepatic cells have been shown to exhibit increased expression of hepatic marker genes and enhanced cell maturation [18]. Furthermore, Okeyo et al. [19] reported that micromesh culture induces spontaneous differentiation of hiPSCs into trophoblast cyst structures without BMP4 treatment.

In the current study, we aimed to establish hiPSC-derived TSCs using the micromesh culture method without BMP4 treatment. We confirmed the differentiation of hiPSC-derived trophoblast cells under micromesh culture conditions and examined whether the cells could differentiate into STBs and EVTs. We hypothesized that micromesh culture techniques may have great potential to yield TSCs from the hiPSCs of patients with infertility or genetic diseases and could facilitate the analysis of trophoblast functions in both normal and diseased pregnancies in vitro. Our findings may establish a potential model for understanding the differentiation and function of trophoblasts and may yield further information regarding the pathogenesis of infertility associated with trophoblast defects for clinical application.

## Methods

### hiPSC culture and differentiation

The ChiPSC22 hiPSC line (Takara Bio, Kusatsu, Japan) was cultured in Cellartis DEF-CS 500 culture medium (Takara Bio) according to the manufacturer’s protocols. Briefly, cells were passaged with TrypLE reagent (Gibco/Life Technologies, Carlsbad, CA, USA) every 3–4 days, and the culture medium was replaced daily. Only passages 10–20 were used for all experiments described here.

**Fig. 1:**
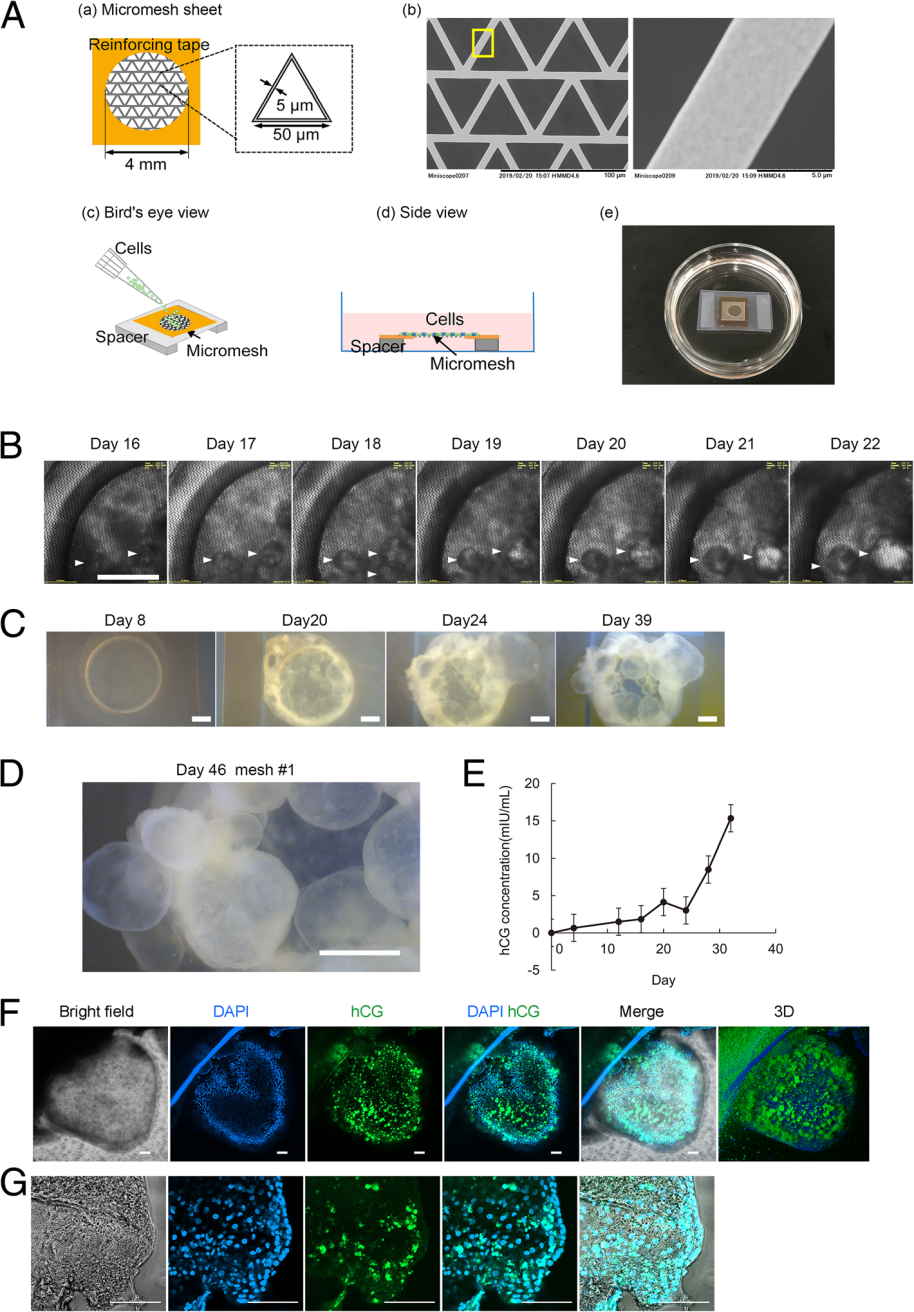
Induction of a trophoblastic cyst structure in hiPSCs in a micromesh culture. **A** Schematic diagram showing the procedures for cell seeding and culture in a limited area on a micromesh. (a) Mesh shapes and dimensions. (b) Scanning electron microscope image of a fabricated triangular mesh. (c, d) Schematic showing how cell seeding and culture were performed on mesh sheets suspended in a culture medium. (e) Real product photograph. A micromesh sheet seeded with hiPSCs was placed in a 3.5-cm dish and cultured for 30–50 days. **B** Time-lapse monitoring of hiPSCs on a 4-mm suspended mesh sheet. Phase-contrast images of hiPSCs on days 16–22 of micromesh culture. Scale bar = 1 mm. **C** Stereomicroscopic images of hiPSCs in micromesh culture. Scale bar = 1 mm. **D** Enlarged view of stereomicroscope images, showing numerous cysts in the micromesh culture on day 46. **E** Quantitative analysis of hCG secretion by ELISA. Culture supernatants were collected from micromesh culture samples. Data are presented as means ± SEMs (*n* = 4). **F** Immunofluorescence analysis of cysts. Bright-field and immunofluorescence images of representative cysts stained for hCG. Nuclei were stained with DAPI (blue). Scale bar = 100 μm. **G** High-magnification images of immunofluorescence for hCG. To scan high-magnification images using a confocal microscope, individual cysts were resected and placed on the cover glass for observation. Scale bar = 100 μm

Differentiation of trophoblasts was performed using the micromesh culture method. Briefly, a nickel micromesh with triangular shapes (Optnics Precision Co., Ltd., Ashikaga, Japan) was obtained. The side length of the triangle was 50 μm, the thickness of the sheet was 5 μm, and the width of the mesh strand was 5 μm (Fig. 1A—a, b). The micromesh was cut into 0.6 × 0.6 cm sections and fixed on a silicon sheet (1 × 0.5 × 1 cm, length × height × width) using a Kapton tape (Fig. 1A—a). The silicon sheet and Kapton tape had a 4-mm hole in the center to act as a frame reinforcing the ultrathin mesh sheets (Fig. 1A—a). The mesh was coated with a thin layer of parylene in a PDS2010 system (Specialty Coating Systems) and was then air plasma treated using a plasma cleaner (PDC-001-HP; Harrick Plasma) at a high radio frequency for 3 min. The reinforced mesh sheets were sterilized using ultraviolet irradiation for 90 min. Following sterilization, 50 μL of 3.2 μg/mL iMatrix-511 (Takara Bio) was applied to the hole of the mesh sheet and allowed to coat the hole at 37 °C for at least 1 h. Next, 50 μL of an hiPSC suspension (2 × 10^6^ cells/mL) was gently pipetted into the hole of the mesh sheet (Fig. 1A—c). After incubation at 37 °C for 4 h, the mesh sheets were suspended in culture medium using a 0.5-cm-thick silicon spacer such that the cells seeded on the mesh sheets were completely out of contact with the dish bottom (Fig. 1A—d, e). hiPSCs were cultured on a mesh sheet at 37 °C in an atmosphere of 5% CO_2_ in DEF-CS medium for approximately 30–50 days, with the culture medium replaced every 3 days.

### TSC culture

After 30–50 days, trophoblast cysts were observed in the mesh culture. The cysts were separated from the mesh using microscissors or a microscalpel (Additional file 3: Movie S1) and transferred to iMatrix-511-coated 24-well plates. Each cyst was seeded in a single well. For proliferation of cystic trophoblasts on the bottom of the plate, the basic culture medium for hTSC reported by Okae et al. [10] was used. Briefly, the cysts were cultured in hTSC medium (Dulbecco’s modified Eagle’s medium [DMEM]/F12 medium supplemented with 0.1 mM 2-mercaptoethanol, 0.2% fetal bovine serum, 1% penicillin/streptomycin, 0.3% bovine serum albumin [BSA], 1% ITS-X supplement, 1.5 mg/mL l-ascorbic acid, 50 ng/mL epidermal growth factor [EGF], 2 mM CHIR99021, 0.5 mM A83-01, 1 mM SB431542, 0.8 mM valproic acid, and 5 mM Y27632). The culture medium was replaced every 2 days.


**Additional file 3: Movie S1.** A single cyst was isolated from a micromesh culture on day 46 of culture. (MP4 3870 kb)


Ten days after cyst seeding, cells proliferating from cysts were collected and passaged. The cells were characterized as TSCs. For passaging, these TSCs were seeded at a density of 2.5 × 10^5^ cells/well in 6-well plates, which was precoated with 1.5 mL of 3.2 μg/mL iMatrix-511, and cultured in 2-mL hTSC medium for 3–4 days, with culture medium replaced every 2 days. At 70–80% confluence, the cells were dissociated with TrypLE and Accumax (1:1) for 6 min at 37 °C and then reseeded and cultured in an hTSC medium. The cells initially grew slowly but then yielded highly proliferative cells within several passages. Thereafter, trophoblast cells were routinely passaged every 3 days at a 1:4 split ratio. For cryopreservation, trophoblast cells were suspended in Cell Banker 1 and stored in a deep freezer at − 80 °C or in liquid nitrogen.

### TSC differentiation

For differentiation of TSCs into EVTs and STBs, a procedure described by Okae et al. [10] was used. Briefly, TSCs were grown to 80% confluence in hTSC medium and dissociated with TrypLE and Accumax (1:1) for 6 min at 37 °C. For induction of three-dimensional (3D) STB cells, TSCs were seeded in low-adhesion Petri dishes at a density of 2.5 × 10^5^ cells/well and cultured in 3-mL STB-(3D) medium (DMEM/F12 supplemented with 0.1 mM 2-mercaptoethanol, 0.5% penicillin/streptomycin, 0.3% BSA, 1% ITS-X supplement, 2.5 mM Y27632, 50 ng/mL EGF, 2 mM forskolin, and 4% knockout serum replacement [KSR]). An equal volume of fresh STB-(3D) medium was added on day 3, and the cells were analyzed on day 6.

For induction of EVTs, TSCs were seeded at a density of 0.75 × 10^5^ cells/well in 6-well plates precoated with 1 mg/mL collagen IV and then cultured in 2-mL EVT medium (DMEM/F12 supplemented with 0.1 mM 2-mercaptoethanol, 0.5% penicillin/streptomycin, 0.3% BSA, 1% ITS-X supplement, 100 ng/mL human neuregulin-1 [NRG1], 7.5 mM A83-01, 2.5 mM Y27632, and 4% KSR). Matrigel was added to a final concentration of 2% shortly after suspending the cells in the medium. On day 3, the medium was replaced with EVT medium without NRG1, and Matrigel was added to a final concentration of 0.5% [10].

### Immunostaining

Cells were fixed with 4% paraformaldehyde (PFA) for 15 min, permeabilized with 0.1% Triton X-100 for 10 min, and blocked with Blocking One buffer (Nacalai Tesque, Kyoto, Japan) for 1 h at room temperature. The cells were then incubated with primary antibodies overnight at 4 °C. The following primary antibodies were used: anti-hCG (cat. no. ab9582; 1:100; Abcam, Cambridge, UK), anti-E-cadherin (cat. no. 3195; 1:100; Cell Signaling Technology, Danvers, MA, USA), anti-tumor protein p63 (TP63; cat. no. 13109; 1:100; Cell Signaling Technology), anti-SRY (sex determining region Y)-box 2 (SOX2; cat. no. 3579S; 1:400; Cell Signaling Technology), anti-Nanog homeobox (NANOG; cat. no. 4903S; 1:200; Cell Signaling Technology), anti-keratin 7 (KRT7; cat. no. 15539-1-AP; 1:200; Proteintech, USA), and anti-major histocompatibility complex, class I, G (HLA-G; cat. no. 4H84; 1:100; Abcam), anti-POU class 5 homeobox 1 (POU5F1; cat. no. 75463; 1:200; Cell Signaling Technology), and anti-GATA-binding protein 3 (GATA3; cat. no. 96098; 1:100; Cell Signaling Technology). Finally, cells were incubated with Alexa Fluor 488- or Alexa Fluor 594-conjugated secondary antibodies (1:500; Invitrogen, Carlsbad, CA, USA). All antibodies were diluted in phosphate-buffered saline (PBS) containing 1% BSA. For hiPSCs, the undifferentiation staining lectin marker rBC2LCN-488 (100-fold diluted; Wako Pure Chemical Industries, Ltd., Osaka, Japan) was mixed with medium, and hiPSCs were incubated in this medium for 2 h at 37 °C and 5% CO_2_ for labeling. Nuclei were stained with 4′,6-diamidino-2-phenylindole (DAPI) or Hoechst 33342, and images were acquired with a laser scanning confocal microscope (LSM 800; Carl Zeiss, Oberkochen, Germany).

### Analysis of hCG

Aliquots of medium were collected for enzyme-linked immunosorbent assay (ELISA) analysis from cultures with hiPSC-derived cysts and stored at − 20 °C. Secreted hCG was assayed using an hCG ELISA kit (Immunospec, Canoga Park, CA, USA) according to the manufacturer’s instructions.

### Real-time quantitative polymerase chain reaction (qRT-PCR) analysis

Cells plated in 24-well plates (5,000 cells/well) or hiPSC-derived cysts were collected, and qRT-PCR was performed using a Power SYBR Green Cells-to-CT kit, as described by the manufacturer (Invitrogen). The expression level of each gene was normalized to that of glyceraldehyde 3-phosphate dehydrogenase (*GAPDH*) used as an internal control. Representative data from three independent experiments are shown. The primers used for qRT-PCR are shown in Additional file 1: Table S1.

### Flow cytometry

Cells were dissociated with TrypLE and Accumax (1:1) for 6 min at 37 °C and resuspended in PBS. For flow cytometric analysis of KRT7, cells were fixed with 4% PFA for 15 min at room temperature and permeabilized with 0.1% Triton X-100 for 10 min. The cells were then incubated with anti-KRT7 antibodies (1:100) overnight at 4 °C and stained with Alexa Fluor 488-conjugated anti-rabbit IgG for 1 h at 25 °C. Normal rabbit IgG was used as an isotype control. Flow cytometry was carried out using an SH800 cell sorter (Sony, Tokyo, Japan), and the data were analyzed using SH800 software.

### mRNA expression array analysis

For mRNA microarray analysis, 3D-Gene Human Oligo Chip 25 K (Toray Industries, Inc., Tokyo, Japan) was used. Total RNA was amplified using an amino allyl aRNA kit (Ambion, Inc., Foster City, CA, USA). The total RNA was labeled with cyanine 5 (Cy5) using Amersham Cy5 mono-reactive dye (GE Healthcare, Little Chalfont, Buckinghamshire, UK). Cy5-labeled aRNAs were individually hybridized at 37 °C for 16 h. The chips were washed and scanned using a 3D-Gene scanner 3000 (Toray Industries, Inc.) and analyzed using 3D-Gene extraction software (Toray Industries, Inc.). The signals detected for each gene were subjected to global normalization (the median of the detected signal intensity was adjusted to 25).

### Statistical analysis

Data are presented as means ± standard errors of the means (SEMs). Statistical analyses were conducted with SPSS Statistics version 22 (IBM, Armonk, NY, USA). Student’s *t* tests were used to determine the significance of differences, which was defined as *P* < 0.05. For changes in hCG secretion by TS^hiPSC^ cells, one-way analysis of variance with repeated measures was conducted.

## Results

### Identification of hCG-positive trophectoderm-like cysts derived from hiPSCs in micromesh culture

Micromesh scaffolds have been used for hiPSC differentiation into trophectoderm-like cysts [19, 20]. Consistent with previously reported data, our data showed that cystic structures emerged on day 16 when using a 4-mm nickel micromesh (Fig. 1B). With continuous culture, the cysts grew to a diameter of ~ 2 mm (Fig. 1B–D).

We then assayed the secretion of hCG, a key pregnancy hormone secreted by placental trophoblasts [21], using ELISAs. The levels of hCG markedly increased in the cyst-containing medium after day 15 (Fig. 1E) and continued to increase up to day 35. The localization of hCG in the cysts was examined by immunostaining, and the results revealed the presence of hCG in the cytoplasm of cyst-forming cells, providing evidence for hiPSC differentiation into cystic trophoblast cells in the micromesh culture (Fig. 1F, G).

### Generation of trophoblast stem-like cells from hiPSC-derived cysts

**Fig. 2:**
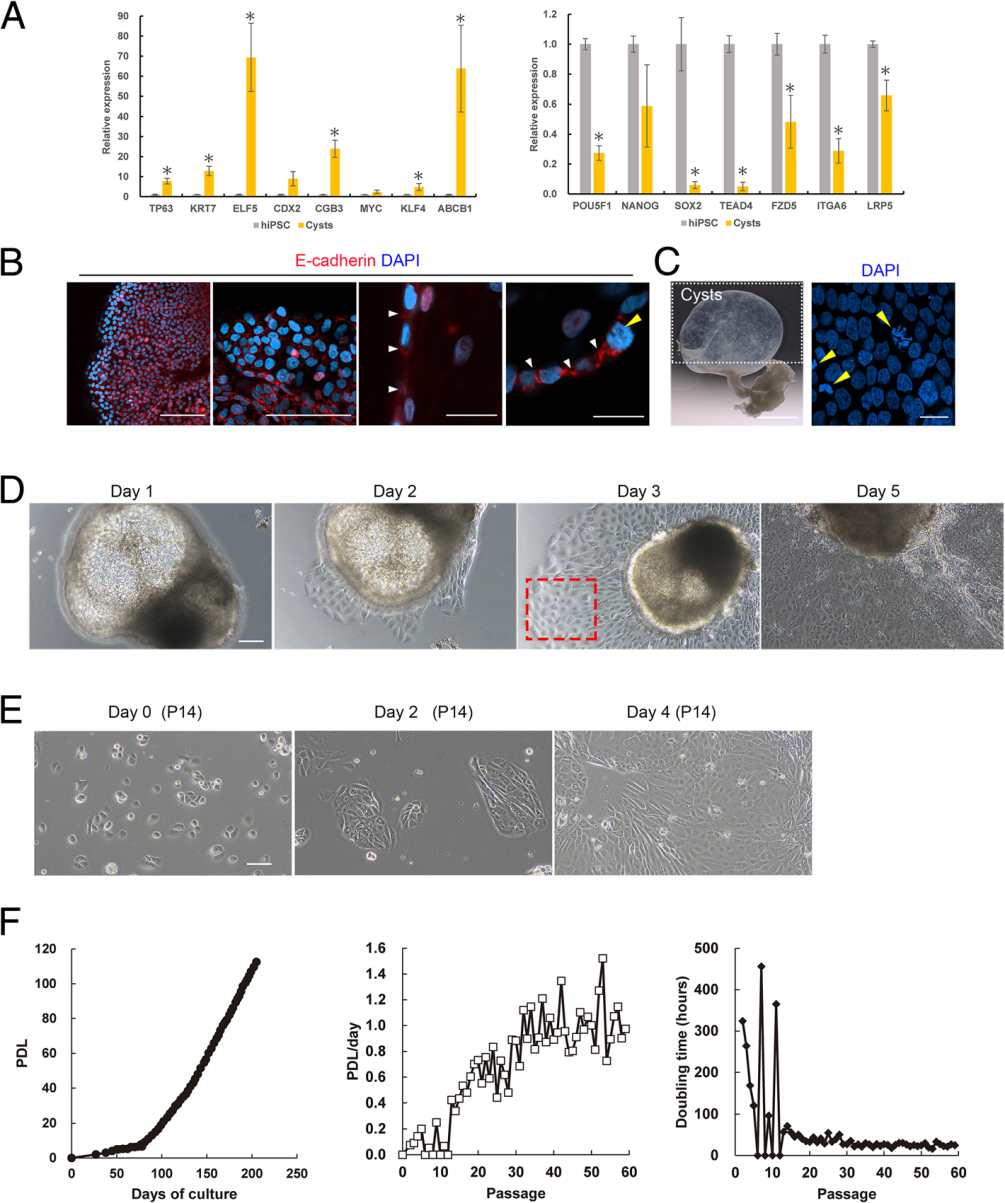
Establishment of proliferative trophoblast cells from hiPSC-induced cysts. **a** Analysis of pluripotency and trophoblast gene expression by qRT-PCR in hiPSCs and cysts. Cysts were collected on day 46, and hiPSCs were grown on laminin-coated dishes for 3 days. Expression levels were calculated relative to those of *GAPDH* and normalized to those of control hiPSCs. Values are the means ± SEMs (*n* = 4). Significance of differences was determined by Student’s *t* tests. *P < 0.05 versus the hiPSC group. **b** Immunofluorescence images of a representative cyst stained for E-cadherin. Scale bar = 100 μm (two left images) and 20 μm (two right enlarged images). Similar results were obtained for three independent samples from separate dishes. **c** Stereomicroscope images of separated cysts from 4-mm micromesh culture on day 46. Left: scale bar = 1 mm. Right: nuclei were stained with DAPI (blue). Scale bar = 20 μm. **d** Phase-contrast images of time-lapse monitoring of a single cyst on days 1–5. The cyst was isolated from the micromesh culture and transferred to 24-well plates on day 0. Cells derived from cysts in the area within the red-dotted box were recovered for reseeding of trophoblast cells. Scale bar = 100 μm. **e** Phase-contrast images showing the growth of trophoblast cells derived from cysts after 14 passages. Scale bar = 100 μm. **f** Proliferation rate of trophoblast cells derived from cysts. Left, population doubling levels (PDLs) versus days of culture; middle, PDL/day versus passage number; and right, doubling time (h) versus passage number

We then analyzed the expression of key pluripotency genes and trophoblast lineage-specific genes in trophectoderm-like cysts using qRT-PCR analysis. The expression levels of *POU5F1* and *SOX2* significantly decreased in cysts compared with that in hiPSCs (Fig. 2a). Moreover, *NANOG* was weakly expressed in the collected cysts, potentially because of the presence of undifferentiated cells.

Expression levels of STB-specific markers, including chorionic gonadotropin subunit beta 3 (*CGB3*) and Kruppel-like factor 4 (*KLF4*), significantly increased in cysts compared with those in hiPSCs (Fig. 2a). Expression levels of hTSC markers, including *TP63*, *KRT7*, and E74-like ETS transcription factor 5 (*ELF5*), significantly increased in cysts compared with those in hiPSCs. ATP-binding cassette subfamily B member 1 (ABCB1) is a widely studied drug efflux transporter in the placental barrier, and its mRNA expression was markedly increased in the cysts compared with those in hiPSCs, indicating the presence of STBs. Caudal type homeobox 2 (*CDX2*) is functional in trophectoderm cells, and its mRNA expression increased 8-fold in cysts compared with that in hiPSCs (*P* = 0.7). Gene expression of MYC proto-oncogene (*MYC*) increased 1.4-fold in cysts compared with that in hiPSCs (*P* = 0.1; Fig. 2a). TEA domain transcription factor 4, frizzled class receptor 5, integrin subunit alpha 6, and low-density lipoprotein receptor-related protein 5 play important roles in the formation and maintenance of hTSCs, and their gene expression levels did not increase in the cysts compared with those in hiPSCs. These results demonstrated that hiPSC-derived trophoblast cysts may contain different types of cells, including STBs, TSCs, and undifferentiated hiPSCs.

Next, the localization of E-cadherin was analyzed in hiPSC-derived cysts by immunostaining. We found that E-cadherin was clearly expressed at the interfaces between mononuclear cells (Fig. 2b). Nuclear staining of cysts with DAPI showed that several cells were undergoing cell division (Fig. 2c). Based on these results, we attempted to obtain proliferating and mononuclear cells from hiPSC-derived cysts.

Cysts were separated and reseeded into cell culture dishes, and cells differentiating from cysts showed growth capacity (Fig. 2d). Because cysts contained several types of cells, we removed the cyst portion of the aggregates from the culture, and cells proliferating from the cysts (red-dotted box, Fig. 2d) were collected on day 3 for reseeding. The collected cells continued to proliferate in the hTSC medium and could be propagated for at least 59 passages, with 113 population doublings over 205 days (Fig. 2e, f). During the first 10 passages, the cells initially grew slowly; however, after several passages, the cells gave rise to highly proliferative cells, with a doubling time of 20–35 h (Fig. 2f). These proliferative trophoblasts were designated hiPSC-derived TSCs (TS^hiPSC^) because they had the ability to differentiate into EVT- and STB-like cells, as detailed below.

### Establishment of TSCs from hiPSC-induced cysts (TS^hiPSC^)

**Fig. 3:**
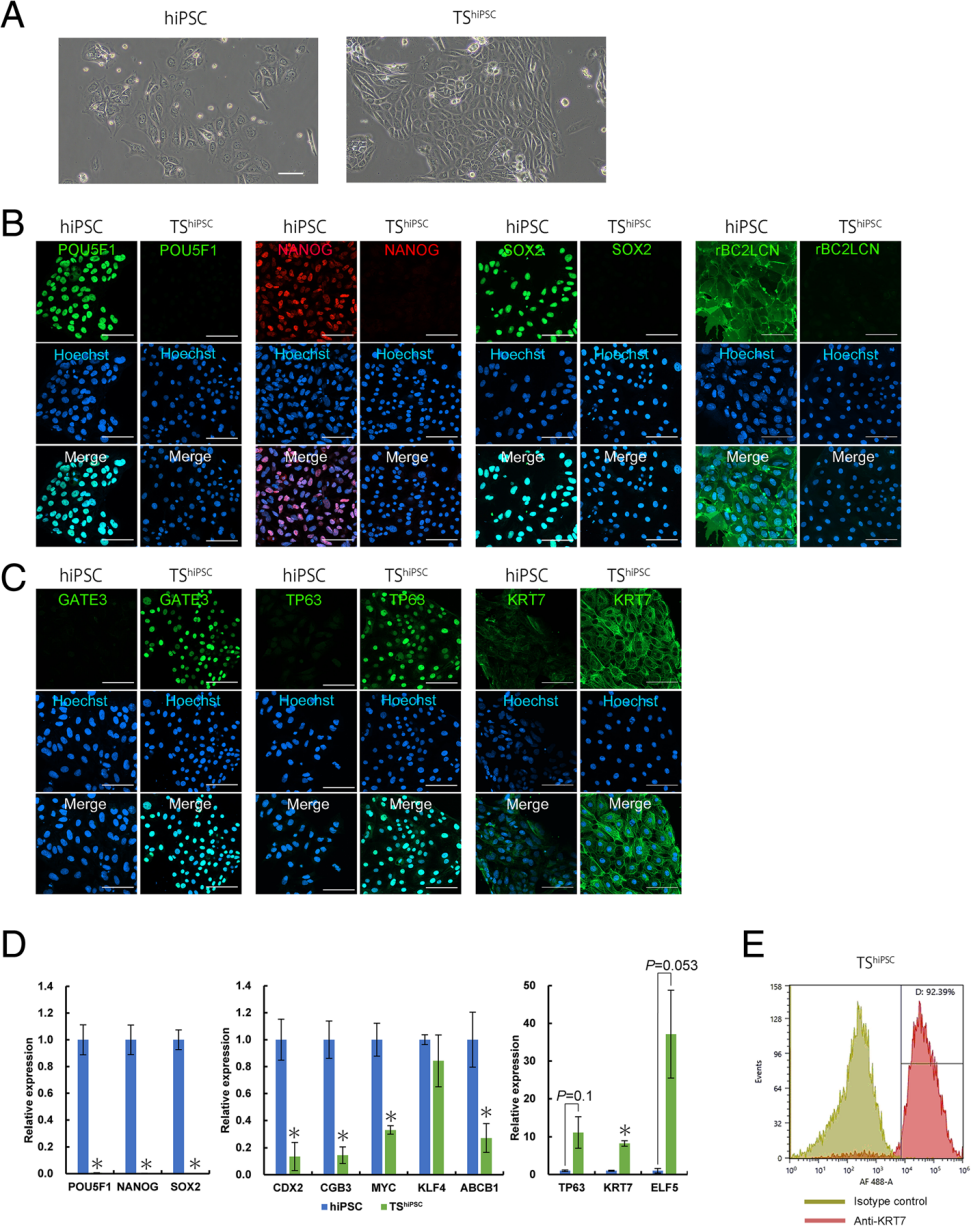
Morphology of and marker expression in hiPSCs and trophoblast cells derived from cysts (TS^hiPSC^). **a** Phase-contrast images of hiPSCs and TS^hiPSC^ cells. Scale bar = 100 μm. **b**, **c** Bright-field and immunofluorescence images of hiPSCs and TS^hiPSC^ cells stained for POU5F1, NANOG, SOX2, and rBC2LCN (**b**) and GATA3, TP63, and KRT7 (**c**). Nuclei were stained with Hoechst 33342. Scale bar = 100 μm. **d** Analysis of pluripotency and trophoblast gene expression by qRT-PCR in hiPSCs and TS^hiPSC^ cells grown on laminin-coated dishes for 3 days. Expression levels were calculated relative to those of *GAPDH* and normalized to those of control hiPSCs. Values are the means ± SEMs (*n* = 4). Significance of differences was determined by Student’s *t* tests. *P < 0.05 versus the hiPSC group. **e** Flow cytometry histograms of KRT7 expression in TS^hiPSC^ cells. Similar results were obtained for three independent cell samples

We next characterized TS^hiPSC^ cells as undifferentiated CTBs. TS^hiPSC^ cells showed a different cell morphology from hiPSCs (Fig. 3a). Compared with undifferentiated hiPSCs, TS^hiPSC^ cells showed loss of the pluripotency markers NANOG, SOX2, and rBC2LCN (Fig. 3b); in contrast, the hTSC-associated proteins GATA3, TP63, and KRT7 were all highly expressed in TS^hiPSC^ cells (Fig. 3c). The results of qRT-PCR analysis showed that there was almost no expression of *POU5F1*, *NANOG*, and *SOX2* in TS^hiPSC^ cells, whereas *TP63* and *KRT7* expression levels were significantly increased in TS^hiPSC^ cells compared with those in hiPSCs (Fig. 3d). In particular, the TSC-associated marker *ELF5* was induced by 36-fold in TS^hiPSC^ cells (Fig. 3d). The STB markers *CGB3*, *MYC*, *KLF4*, and *ABCB1* decreased in TS^hiPSC^ cells (Fig. 3d). The level of cell purity was assessed by measuring the intracellular expression of the pan-trophoblast marker KRT7 (Fig. 3e). The purity of KRT7-positive cells was greater than 90% (Fig. 3e). These data indicated that cells derived from hiPSC-induced cysts assumed an hTSC phenotype. Furthermore, hTSC-associated marker expression in TS^hiPSC^ cells after several passages was assessed by immunostaining and qRT-PCR analysis. The hTSC-associated proteins KRT7, TP63, and GATA3 were all highly expressed in TS^hiPSC^ cells after 15, 25, 35, and 55 passages (Additional file 4: Figure S1A). The results of qRT-PCR analysis showed that the genes *TP63 GATE3*, *KRT7*, and *ELF5* were also all highly expressed in TS^hiPSC^ cells after 15, 25, 35, 45, and 55 passages compared with those in hiPSCs (Additional file 4: Figure S1B). The expression levels of these hTSC-associated markers were sustained after several passages.

### Global gene expression profiling of hiPSCs and TS^hiPSC^ cells

**Fig. 4:**
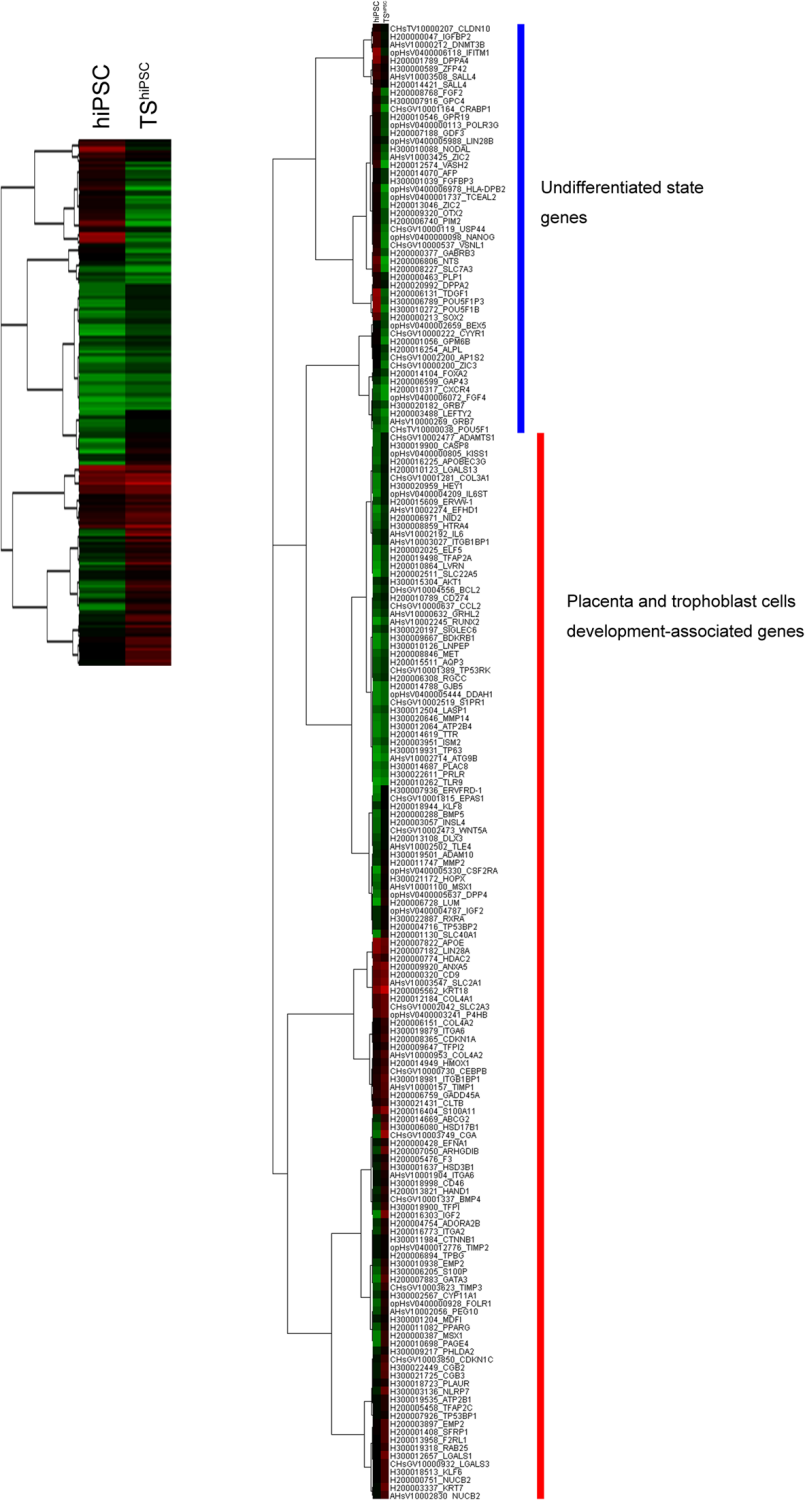
Comparison of gene signatures between hiPSCs and TS^hiPSC^ cells. Hierarchical clustering heat-map based on the expression of mRNA extracted from hiPSCs and TS^hiPSC^ cells. Right heat-map including gene names. Green, downregulated genes; red, upregulated genes

To improve our understanding of the molecular characteristics of TS^hiPSC^ cells, we used a microarray approach, with hiPSCs as a control group. Trophoblast- and hiPSC-related genes are shown in Fig. 4, and the entire data set and full gene names are included in Additional file 2: Table S2. Compared with their expression in hiPSCs, pluripotency factors, including *AFP*, *DNMT3B*, *DPPA2*, *DPPA4*, *FGF2*, *FOXA2*, *GDF3*, *GRB7*, *LEFTY2*, *LIN28B*, *NANOG*, *POU5F1*, *SOX2*, *ZFP42*, and *ZIC2*, were downregulated in TS^hiPSC^ cells.

Genes responsible for trophoblast differentiation and function were expected to be upregulated in TS^hiPSC^ cells. Several genes upregulated in Fig. 4 have previously been shown to be involved in trophoblast development, such as genes encoding collagens (*COL4A1* and *COL4A2*), keratins (*KRT7* and *KRT18*), calcium-binding proteins (*S100A11* and *S100P*), a transporter (*SLC40A1*), hormones (*CGA* and *CGB*), and components of the extracellular matrix (*COL3A1* and *NID2*). Others included the *GATA3* gene, which is an important in vivo regulator of trophoblast-specific gene expression and placental function [22]; the *HSD3B1* gene, which is necessary for the biosynthesis of placental progesterone and is thus essential for pregnancy maintenance [23]; the placenta-specific *IGF2* gene, which is a major modulator of placental and fetal growth [24]; and the *EPAS1* gene, which is a hypoxia-responsive transcription factor involved in the regulation of endothelial cell gene expression [25]. *KRT7*, *GATA3*, and *TFAP2C*, which are good markers for first-trimester mononuclear trophoblast cells [26], were upregulated in TS^hiPSC^ cells. *CDKN1C*, *CSF2RA*, *CYP11A1*, *EFHD1*, *FOLR1*, *HTRA4*, *ISM2*, *LVRN*, *PAGE4*, and *TFAP2C* genes, which are expressed in primary CTBs from second-trimester placentas [27], were also upregulated in TS^hiPSC^ cells compared with those in hiPSCs. Moreover, microarray analysis confirmed the changes in gene expression profiles, as revealed by qRT-PCR analysis (Fig. 3d).

In this study, TS^hiPSC^ cells were induced from hiPSCs in a micromesh culture without BMP treatment. However, several genes associated with the emergence of trophoblast cells from BMP-treated hESCs, such as *HAND1*, *DLX3*, *CGA*, *CGB*, *IGF2*, *CYP11A1*, *HSD17B1*, *COL4A1*, *COL4A2*, *KRT7*, *KRT18*, *MMP2*, *TIMP3*, *S100A11*, *S100P*, *SLC40A1*, *PPARG*, and *HOPX* [28–30], were upregulated in TS^hiPSC^ cells. Therefore, we also evaluated the transforming growth factor-β superfamily signaling network, including the BMP/GDF and ACTIVIN/NODAL branches. Inhibition of ACTIVIN/NODAL signaling and activation of BMP signaling are required for trophoblast differentiation from hESCs [31]. The microarray analysis showed systematic activation of directly inducible BMP target genes (*DLX3*, *GATA3*, *HEY1*, and *MSX1*). Genes encoding ligands and receptors supporting BMP signaling (*BMP4*, *BMP5*, and *BMP7*) were also induced. In contrast, genes encoding ACTIVIN/NODAL ligands (*FGF2*, *FGF4*, and *NODAL*) [31] were downregulated. Similarly, the *GDF3* gene, a known pluripotency-associated marker, acting via the initial signaling proteins SMAD2/3 of ACTIVIN/NODAL branches, was downregulated. Taken together, these gene expression pattern results provided reliable evidence for the characterization of TS^hiPSC^ cells as assumptive hTSCs.

### Differentiation capacity of TS^hiPSC^ cells

**Fig. 5:**
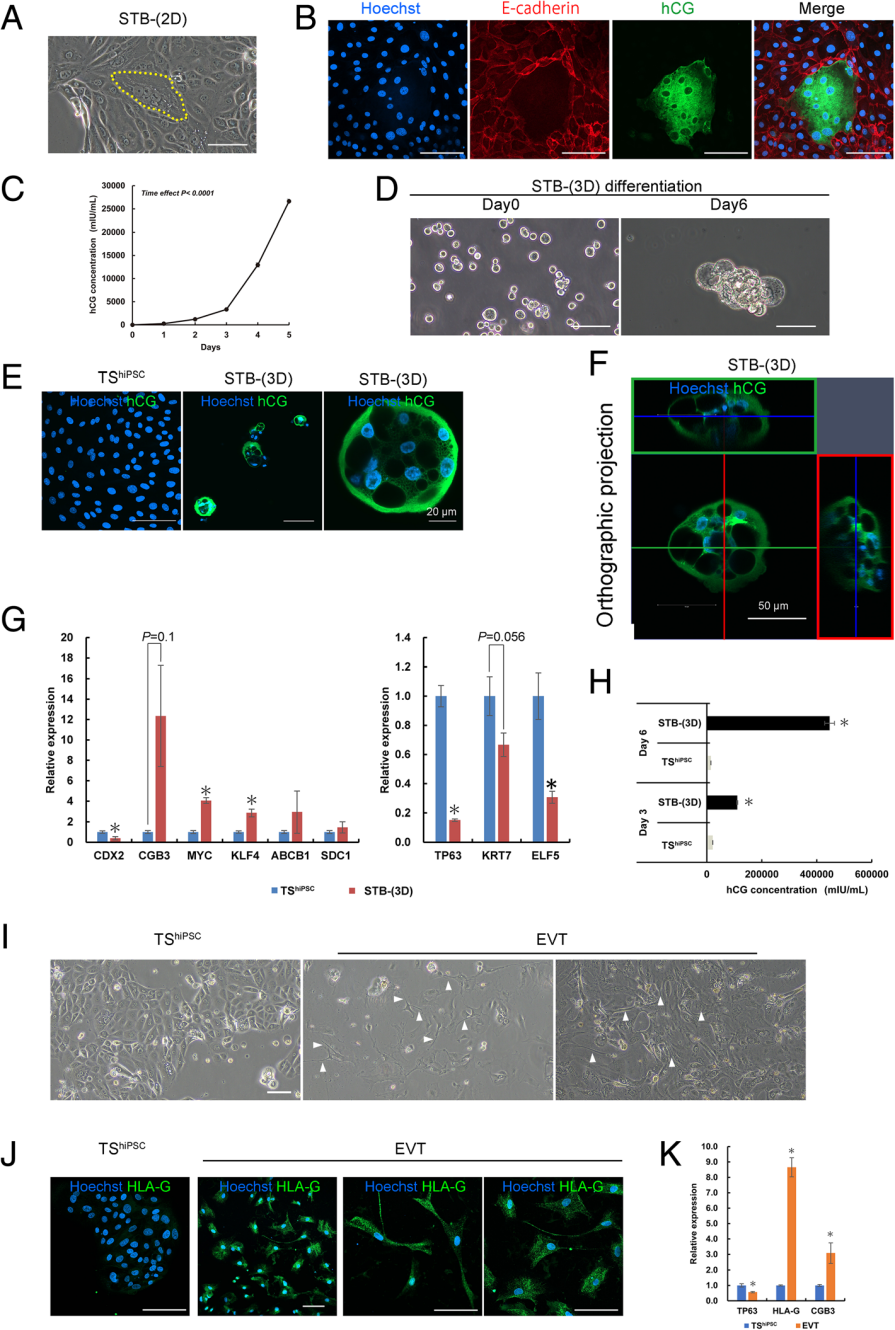
Directed differentiation of TS^hiPSC^ cells into STB- and EVT-like cells. **a** Phase-contrast image of TS^hiPSC^ cells containing multinucleated STB-(2D) cells (yellow-dotted line area). **b** Immunofluorescence images of TS^hiPSC^ cells containing multinucleated STB-(2D) cells stained for E-cadherin and hCG; nuclei were stained with Hoechst 33342. **c** Changes in hCG secretion by TS^hiPSC^ cells over 5 days. Data are presented as means ± SEMs (*n* = 4). Significance of differences was determined by one-way analysis of variance with repeated measures. **d** Phase-contrast images of STB-(3D) cells in low-adherence culture dishes on days 0 and 6. **e** Immunofluorescence images of TS^hiPSC^ cells and STB-(3D) cells stained for hCG. **f** Immunofluorescence images of STB-(3D) cells at *z*-position, showing the STB-(3D) cyst structure, which had several clear interior cavities and a thin enclosing wall. **g** Analysis of pluripotency and trophoblast gene expression by qRT-PCR in TS^hiPSC^ and STB-(3D) cells. Expression levels were calculated relative to those of *GAPDH* and normalized to those of TS^hiPSC^ cells. Values are the means ± SEMs (*n* = 4). Significance of differences was determined by Student’s *t* tests. *P < 0.05 versus the TS^hiPSC^ cell group. **h** Levels of hCG secreted by TS^hiPSC^ and STB-(3D) cells. For comparison, both TS^hiPSC^ and STB-(3D) cells were cultured in low-adherence Petri dishes. Data are presented as means ± SEMs (*n* = 3). **i** Phase-contrast images of TS^hiPSC^ and EVT cells. EVT cells had a mesenchyme-like morphology. The arrows indicate the presence of pseudopodia on EVT cells. **j** Immunofluorescence images of TS^hiPSC^ and EVT cells stained for HLA-G. **k** Analysis of *TP63*, *HLA-G*, and *CGB* gene expression by qRT-PCR in TS^hiPSC^ and EVT cells. Expression levels were calculated relative to those of *GAPDH* and normalized to those in TS^hiPSC^ cells. Values are the means ± SEMs (*n* = 4). Significance of differences was determined by Student’s *t* tests. *P < 0.05 versus the TS^hiPSC^ cell group. Unless otherwise noted, the scale bars in all phase-contrast and immunostaining images are 100 μm

To determine whether TS^hiPSC^ cells were multipotent, we investigated their ability to terminally differentiate into both STBs and EVTs. Some TS^hiPSC^ cells cultured in the hTSC medium spontaneously fused to form syncytia (Fig. 5a). The syncytium cells were designated STB-(2D) cells. Immunostaining for E-cadherin clearly showed that the STB-(2D) cells were multinuclear (Fig. 5b). The STB marker hCG was highly expressed in these multinuclear syncytia (Fig. 5b). In addition, we observed that higher cell densities were associated with greater numbers of syncytia. The ELISA results showed an increase in hCG levels in 5-day plate culture (Fig. 5c).

Previous studies have reported that cAMP enhances STB formation [32]. Therefore, we treated TS^hiPSC^ cells with a cAMP agonist, forskolin. However, unlike hTSCs derived from placenta tissue [10], these cells did not effectively fuse to form large syncytia in iMatrix-511-coated dishes. Notably, 3D culture has been reported to enhance the differentiation of trophoblast cells into STB-like cells [8]. Thus, we cultured TS^hiPSC^ cells in low-adhesion Petri dishes and observed that the cells formed cyst-like cells after 6 days of differentiation (Fig. 5d). The immunostaining results showed the expression of hCG in the cysts (Fig. 5e, f). These cyst-like cells were designated STB-(3D) cells. Based on the qRT-PCR analysis, the expression levels of the hTSCs markers *TP63*, *KRT7*, and *ELF5* decreased, and those of STB-related markers *CGB*, *MYC*, *KLF4*, *ABCB1*, and syndecan 1 (*SDC1*) increased in STB-(3D) cells (Fig. 5g). The ELISA results showed that the levels of hCG secreted by STB-(3D) cells were significantly higher than those secreted by TS^hiPSC^ cells (Fig. 5h). The differentiation capacity of TS^hiPSC^ cells into STB-like cells was demonstrated at passage 59 (Additional file 5: Figure S2A–C).

In addition, we examined the differentiation ability of TS^hiPSC^ cells into EVT-like cells using a Matrigel-supplemented medium, as described above. The differentiated cells became morphologically different on day 6 (Fig. 5i). The presence of pseudopodia and a mesenchyme-like morphology were noted in these cells (Fig. 5i). HLA-G expression is a known marker for the identification of EVT subpopulations [33]. Immunostaining confirmed HLA-G expression in these differentiated cells (Fig. 5j). Moreover, the qRT-PCR results showed that the human TS marker *TP63* decreased, whereas *HLA-G* and *CGB* expression levels were significantly increased in these differentiated cells compared with their expression in TS^hiPSC^ cells (Fig. 5k). Taken together, based on both marker expression and function, these data confirmed the bipotential differentiation ability of TS^hiPSC^ cells.

## Discussion

In this study, we successfully purified proliferative CTB-like cells derived from trophectoderm-like cysts, which were induced using a micromesh culture technique, and characterized their ability to proliferate and differentiate as TSCs. Lee et al. [26] suggested that the expression levels of GATA3 and KRT7 proteins should be evaluated as robust criteria for the identification of human first-trimester mononuclear trophoblasts. Importantly, forced expression of TP63 maintains the undifferentiated state of primary CTBs isolated from human first-trimester placental tissues [1]. TP63 expression is significantly downregulated in the later trophoblast lineages EVTs and STBs [1, 10]. Furthermore, both HLA-G and hCG are known to define the two main trophoblast differentiation pathways, i.e., EVTs and STBs, respectively [26]. Our TS^hiPSC^ cells met all the above criteria and had the ability to directly differentiate into HLA-G-positive EVTs or hCG-secreting STBs. Our comprehensive data strongly suggested that TS^hiPSC^ cells may reproduce the features of early hTSCs.

Previous reports by Okeyo et al. [19, 20] have shown that limited culture on micromesh can induce hiPSCs to differentiate into trophoblast cysts. Unlike the studies by Okeyo et al. [19, 20], in this study, we used a commercially available nickel micromesh for the differentiation of hiPSCs. In addition, hiPSC lines and their maintenance media were commercially available. Consequently, we showed the ability of hiPSCs to differentiate into hCG-secreting cysts on the nickel micromesh. Although Okeyo et al. reported that micromesh culture could induce the formation of hCG-secreting cysts, there have been no detailed analyses of the types of cells constituting cysts.

hCG is the most commonly used marker for the identification of STBs [26, 27], and expression of hCG can be used to indicate the presence of STBs in cysts. Immunostaining for E-cadherin showed cell/cell contacts between individual cells in cysts, demonstrating that cysts were primarily composed of mononuclear cells. Thus, we propose that when the micromesh culture method is used, hiPSCs first differentiate into mononuclear CTBs, and these CTBs then further differentiate into STBs in the 3D cystic structure. Interestingly, CTB-like cells were proliferative in hiPSC maintenance medium.

Studies of trophoblast differentiation from hiPSCs and hESCs have focused on the use of BMP4. Short-term (2–6 days) and high-dose (10 ng/mL) BMP4 treatment has been shown to induce a trophoblast lineage [15]. BMP4-induced trophoblast cells often do not really resemble primary CTBs because the composition of the culture medium is inadequate. Recently, two groups have improved BMP4 treatment conditions and confirmed the generation of CDX2-, TP63-, and KRT7-positive CTB populations [1, 15]. These cells could further differentiate into STBs and EVTs upon BMP4 treatment [1, 15]. However, these BMP4-treated CTBs did not have proliferative ability, and their directional differentiation has not been tested [15]. In this study, we used marker analysis, molecular pattern analysis, and secretory function to confirm that the phenotype of TS^hiPSC^ cells was similar to that of BMP4-treated CTBs. In our study, hiPSC-derived proliferative and bipotential CTB-like cells were obtained for the first time. The TS^hiPSC^ cells, as a primary CTB model, were superior to BMP4-treated CTB-like cells.

Identification of BMP4-induced trophoblasts has focused on the induction of CDX2, a transcription factor proposed to be essential for trophectoderm identity and maintenance [34]. CDX2 is detectable until the trophectoderm can be morphologically distinguished and cannot be detected at the morula and early blastocyst stages (~ day 4). CDX2 is then downregulated and translocated to the cytoplasm in human peri-implantation embryos (day 8) [35]. CDX2 is expressed in the human placenta during early gestation (6 weeks) [15] but is expressed at very low levels in hTSCs established from the first-trimester placenta (~ 14 weeks) [10]. Our results showed that expression of the *CDX2* gene was 9-fold higher in trophectoderm-like cysts than in hiPSCs, whereas its level of expression in TS^hiPSC^ cells was only approximately 10% of that in hiPSCs. Moreover, immunostaining results showed that CDX2 protein was barely expressed both in hiPSCs and TS^hiPSC^ cells (Additional file 4: Figure S1C). Quantitative results from immunostaining analyses showed that there were no significant differences between hiPSCs and TS^hiPSC^ cells (Additional file 4: Figure S1D). These results could be related to the observation that some cyst cells were in a differentiated state. When the medium was changed from the hiPSC medium to the hTSC medium, purified TS^hiPSC^ cells showed a homogeneous phenotype, which was similar to that of hTSCs from blastocysts and the first-trimester placenta (TS^placenta^) with low CDX2 expression [10].

Bipotent CTB subtypes are only present in the first-trimester placenta and are lost during the second trimester of pregnancy [36]. CTBs derived from the first-trimester placenta are highly proliferative and have the capacity to generate new villi; these CTBs were suggested to serve as the source of hTSCs [37]. Recently, Okae et al. [10] have successfully isolated and established TS^placenta^. The use of tissues from human embryos and the first-trimester placenta is a good strategy; however, the tissues are often difficult to obtain because of moral and ethical issues. TS^placenta^ cells can proliferate and differentiate into EVT- and STB-like cells [10]. Our TS^hiPSC^ cells, unlike conventional BMP4-treated CTB-like cells, maintained the properties of TS^placenta^ cells. Regarding their ability to differentiate, TS^placenta^ cells can fuse into STBs in 2D culture dishes upon forskolin stimulation. TS^hiPSC^ cells do not efficiently fuse into STBs in 2D culture, but can fuse into 3D-STBs in low-adherence culture. This fusion ability may depend on the adherence ability of TS^hiPSC^ cells. Further studies are needed to determine the fusion conditions for TS^hiPSC^ cells in 2D culture.

In this study, transcriptome analysis was performed on TS^hiPSC^ cells and hiPSCs, and our future research will focus on further identification of differences between TS^hiPSC^ cells and primary trophoblast cells. The ChiPSC22 line used in this study and the hiPSCs (clone TIG1-4F hiPSC from lung fibroblasts, provided by Dr. Takashi Tada of Kyoto University, Japan), which were reported by Okeyo et al. [19, 20], have been shown to be able to differentiate into trophectoderm under micromesh culture. Furthermore, we also found that differentiation into trophectoderm was achieved by spontaneous self-organization, which may or may not occur with similar efficiencies in other hiPSC lines. We used 201B7 and 253G1 hiPSC lines. The 201B7 line only could differentiate to obtain very few cysts (data not shown). The 253G1 line could not differentiate into cysts under the micromesh culture (data not shown).

It is important to determine why the differentiation ability into the trophoblast lineage was different between hiPSCs for application of the micromesh culture technique in the cell differentiation induction field. We also speculated that the production method of hiPSCs, hiPSC properties, and medium components may affect the ability of the cells to differentiate. In the future, we will induce differentiation into trophoblast cells with multiple hiPSC strains and clarify the factors that modulate the induction of differentiation into trophoblast lineages by micromesh culture at a molecular level.

Overall, compared with previous studies on the differentiation of trophoblast cells from hiPSCs, we obtained hTSC-like cells derived from hiPSCs, for the first time. This differentiation process was performed without using BMP4. Therefore, we expect that this simple, versatile micromesh culture technique may have potential applications in the differentiation of iPSCs into other cell types. Horii et al. [15] reported a two-step protocol for obtaining CTB stem-like cells from hiPSCs. However, these CTB stem-like cells were not proliferative, and their redifferentiation could not be controlled directionally. Thus, we propose that, except in primary cells, our micromesh culture protocol may be superior to other currently available models for studying TSCs.

## Conclusions

In summary, we established hTSC-like cells derived from hiPSCs, which were induced using a micromesh technique without any chemical stimulation. These established cells represent a novel, powerful model for studying the function and molecular characteristics of human trophoblast cells. This approach also provided novel insights into the mechanisms of hiPSC differentiation into trophoblast cells and a new experimental system for studying the steps of human trophoblast differentiation. The experimental system may provide a useful tool for understanding the pathogenesis of developmental disorders with trophoblast defects, such as preeclampsia, miscarriage, and intrauterine growth restriction.

## Additional file

Additional file 1: **Table S1.** Markers and primer sequences used for real-time qRT-PCR analysis. (DOCX 15 kb)

Additional file 2: **Table S2.** Global gene expression profiling data. (XLSX 106 kb)

Additional file 4: **Figure S1.** Trophoblast marker expression in trophoblast cells derived from cysts (TS^hiPSC^) after prolonged cell culture. (A) Immunofluorescence images of hiPSCs and TS^hiPSC^ cells stained for KRT7, TP63, and GATA3. Nuclei were stained with Hoechst 33342. Scale bar = 20 μm. (B) Analysis of pluripotency and trophoblast gene expression by qRT-PCR in hiPSCs and TS^hiPSC^ cells. Expression levels were calculated relative to those of *GAPDH* and normalized to those of control hiPSCs. The relative expression levels were also log-transformed. Values are the means ± SEMs (*n* = 4). (C) Immunofluorescence images of hiPSCs and TS^hiPSC^ cells stained for CDX2. Scale bar = 20 μm. (D) Quantitative results of CDX2 expression from immunostaining analyses. Values are the means ± SEMs (*n* = 6). (TIF 6182 kb)

Additional file 5: **Figure S2.** Directed differentiation of TS^hiPSC^ cells (P59) into STB-(3D) cells. (A) Phase-contrast images of TS^hiPSC^ and STB-(3D) cells on days 0, 3, and 5. For comparison, both TS^hiPSC^ and STB-(3D) cells were cultured in low-adherence Petri dishes. (B) High-magnification phase-contrast and stereomicroscope images of STB-(3D) cells on day 5. (C) Immunofluorescence images of TS^hiPSC^ and STB-(3D) cells on day 6. The cells were stainted for hCG, and the nuclei were stained with Hoechst 33342. Scale bar = 100 μm. (TIF 8908 kb)

## Data Availability

The datasets used and/or analyzed during the current study are available from the corresponding author on reasonable request.

## Abbreviations

ABCB1: ATP-binding cassette subfamily B member 1
BMP4: Bone morphogenetic protein-4
cAMP: Cyclic adenosine monophosphate
CDX2: Caudal type homeobox 2
CGB3: Chorionic gonadotropin subunit beta 3
CTBs: Mononuclear cytotrophoblasts
ELF5: E74-like ETS transcription factor 5
EVTs: Extravillous trophoblasts
FGF2: Fibroblast growth factor-2
GAPDH: Glyceraldehyde 3-phosphate dehydrogenase
GATA3: GATA-binding protein 3
hCG: Human chorionic gonadotrophin
hESCs: Human embryonic stem cells
hiPSCs: Human induced pluripotent stem cells
HLA-G: Major histocompatibility complex, class I, G
hTSCs: Human trophoblast stem cells
KLF4: Kruppel-like factor 4
KRT7: Keratin 7
NANOG: Nanog homeobox
PFA: Paraformaldehyde
POU5F1: POU class 5 homeobox 1
qRT-PCR: Real-time quantitative polymerase chain reaction
SOX2: SRY-box 2
STB: Syncytiotrophoblast
TP63: Tumor protein p63
TSCs: Trophoblast stem cells
TS^hiPSC^: TSCs from hiPSC-induced cysts
