# Supplementary material for: Establishment of human trophoblast stem cells from human induced pluripotent stem cell-derived cystic cells under micromesh culture
Source: Stem Cell Res Ther. 2019 Aug 7;10:245. doi: 10.1186/s13287-019-1339-1 (PMC6686486; doi:10.1186/s13287-019-1339-1)
Supplement: Supplementary file 2 — Table S1. Markers and primer sequences used for real-time qRT-PCR analysis. (DOCX 15 kb) [file 13287_2019_1339_MOESM1_ESM.docx]

**Additional file 1:** **Table S1.** Markers and primer sequences used for real-time qRT-PCR analysis.

| Gene names | Forward primer | Reverse primer | Accession |
| --- | --- | --- | --- |
| TP63 | AGAAACGAAGATCCCCAGATGA | CTGTTGCTGTTGCCTGTACGTT | NM_001329964.1 |
| KRT7 | GCGTGAGTACCAGGAACTCATG | GCTTGCGGTAGGTGGCG | XM_011538325.1 |
| ELF5 | TGCCCTCACGGTAATGTTGGA | TGATGCTCAAAGGCAGGGTAG | NM_001243080.1 |
| CDX2 | GGAGCTGGAGAAGGAGTTTCA | TGCAACTTCTTCTTGTTGATTTTC | NM_001265.5 |
| CGB3 | CAGCATCCTATCACCTCCTGGT | CTGGAACATCTCCATCCTTGGT | NM_000737.3 |
| MYC | AAAGGCCCCCAAGGTAGTTA | GCACAAGAGTTCCGTAGCTG | NM_002467.5 |
| KLF4 | GAACTGACCAGGCACTACCG | TTCTGGCAGTGTGGGTCATA | NM_001314052.1 |
| ABCB1 | GCTCCTGACTATGCCAAAGC | ATTAGGCCTTCCGTGCTGTA | NM_001348945.1 |
| POU5F1 | TATTCAGCCAAACGACCATCT | TCAGCTTCCTCCACCCACTT | NM_203289.5 |
| NANOG | ACATGCAACCTGAAGACGTGTG | CATGGAAACCAGAACACGTGG | XM_011520850.1 |
| SOX2 | AGTCTCCAAGCGACGAAAAA | TTTCACGTTTGCAACTGTCC | NM_003106.3 |
| TEAD4 | GGACACTACTCTTACCGCATCC | TCAAAGACATAGGCAATGCACA | NM_201443.2 |
| FZD5 | CCTAAGGTTGGCGTTGTAATG | ACAACTTCCCAGTCACAGCA | NM_003468.3 |
| ITGA6 | AGTGGAGCTGTGGTTTTGCT | AGACCTTCCCCGTCAAAAAT | XM_017004008.1 |
| LRP5 | TGGCCCGAAACCTCTACTG | GCACACTCGATTTTAGGGTTCT | XR_001747874.1 |
| HLA-G | CCACCACCCTGTCTTTGACTAT | ACGTCCTGGGTCTGGTCCT | [NM_001363567.1](https://www.ncbi.nlm.nih.gov/nucleotide/NM_001363567.1?report=genbank&log$=nucltop&blast_rank=1&RID=5R5KTG0V016) |
| SDC1 | CTATTCCCACGTCTCCAGAACC | GGACTACAGCCTCTCCCTCCTT | NM_002997.5 |
| GATE3 | ACATGCTGACCACGCCCACG | GCAGGGCTCTAACCCATGGC | NM_001002295.2 |
| GAPDH | CCTCAACGACCACTTTGTCAAG | TCTTCCTCTTGTGCTCTTGCTG | NM_002046.6 |
