# Supplementary figures and images for: Establishment of human trophoblast stem cells from human induced pluripotent stem cell-derived cystic cells under micromesh culture
Source: Stem Cell Res Ther. 2019 Aug 7;10:245. doi: 10.1186/s13287-019-1339-1 (PMC6686486; doi:10.1186/s13287-019-1339-1)

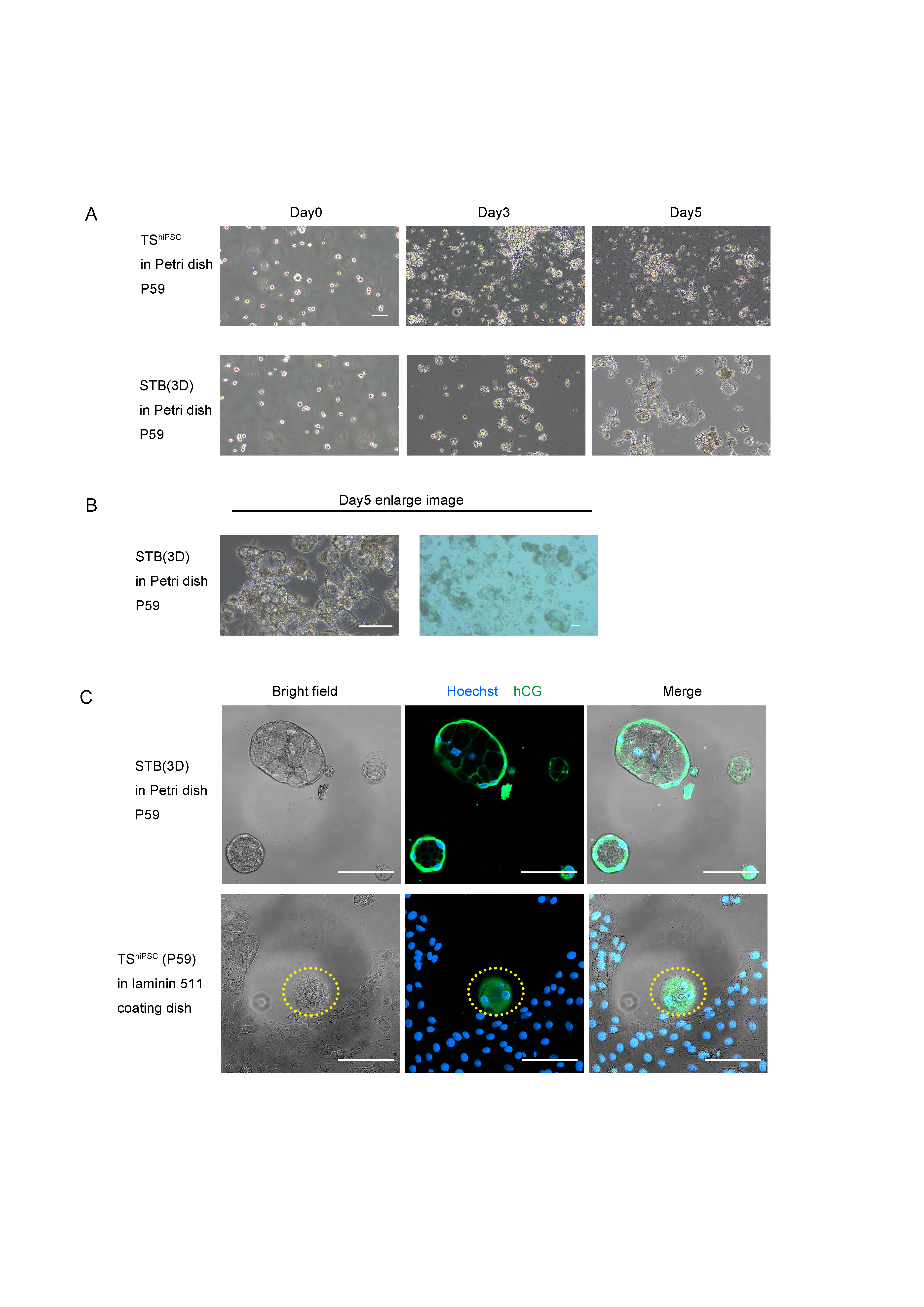

Supplement: Supplementary file 5 — Figure S2. Directed differentiation of TShiPSC cells (P59) into STB-(3D) cells. (A) Phase-contrast images of TShiPSC and STB-(3D) cells on days 0, 3, and 5. For comparison, both TShiPSC and STB-(3D) cells were cultured in low-adherence Petri dishes. (B) High-magnification phase-contrast and stereomicroscope images of STB-(3D) cells on day 5. (C) Immunofluorescence images of TShiPSC and STB-(3D) cells on day 6. The cells were stainted for hCG, and the nuclei were stained with Hoechst 33342. Scale bar = 100 μm. (TIF 8908 kb) [file 13287_2019_1339_MOESM5_ESM.tif]
